# Added value of double reading in diagnostic radiology,a systematic review

**DOI:** 10.1007/s13244-018-0599-0

**Published:** 2018-03-28

**Authors:** Håkan Geijer, Mats Geijer

**Affiliations:** 1 Department of Radiology, Faculty of Medicine and Health, Örebro University, 701 85 Örebro, Sweden; 2 Department of Radiology, Skåne University Hospital and Lund University, Lund, Sweden

**Keywords:** Diagnostic errors, Observer variation, Diagnostic imaging, Review, Quality assurance, healthcare

## Abstract

**Objectives:**

Double reading in diagnostic radiology can find discrepancies in the original report, but a systematic program of double reading is resource consuming. There are conflicting opinions on the value of double reading. The purpose of the current study was to perform a systematic review on the value of double reading.

**Methods:**

A systematic review was performed to find studies calculating the rate of misses and overcalls with the aim of establishing the added value of double reading by human observers.

**Results:**

The literature search resulted in 1610 hits. After abstract and full-text reading, 46 articles were selected for analysis. The rate of discrepancy varied from 0.4 to 22% depending on study setting. Double reading by a sub-specialist, in general, led to high rates of changed reports.

**Conclusions:**

The systematic review found rather low discrepancy rates. The benefit of double reading must be balanced by the considerable number of working hours a systematic double-reading scheme requires. A more profitable scheme might be to use systematic double reading for selected, high-risk examination types. A second conclusion is that there seems to be a value of sub-specialisation for increased report quality. A consequent implementation of this would have far-reaching organisational effects.

**Key Points:**

*• In double reading, two or more radiologists read the same images.*

*• A systematic literature review was performed.*

*• The discrepancy rates varied from 0.4 to 22% in various studies.*

*• Double reading by sub-specialists found high discrepancy rates.*

**Electronic supplementary material:**

The online version of this article (10.1007/s13244-018-0599-0) contains supplementary material, which is available to authorised users.

## Introduction

In the industrialised world, there is an increasing demand for radiology resources with an increasing number of images being produced, which has led to a relative scarcity of radiologists. With limited resources, it is important to question and evaluate work routines, to provide settings for high-quality output and high cost-effectiveness, but at the same time keep medical standards high and avoid costly lawsuits. One way to increase the quality of radiology reports may be double reading of studies between peers, i.e. two radiology specialists of similar and appropriate experience reading the same study.

Most radiologists hold a very firm view on the concept of double reading—either for or against. Arguments for are that it reduces errors and increases quality in radiology. Arguments against are that it does not increase quality significantly, is time-consuming, and wastes time and resources. Despite these firm beliefs, there is comparatively scant evidence supporting either view, and both systems are widely practiced [1]. In some radiology departments or department sections, it is accepted that no systematic double reading is performed between specialists of a similar or above a certain degree of expertise. In other departments, such double reading between peers is mandatory. A survey among Norwegian radiologists reported a double reading rate of 33% of all studies [1], which is consistent with a previous Norwegian survey [2].

The concept of observer variation in radiology was introduced in the late 1940’s when tuberculosis screening with mass chest radiography was evaluated [3, 4]. In a comparison between four different image types (35-mm film, 4 × 10-inch stereophotofluorogram, 14 × 17-inch paper negative, 14 × 17-inch film), it was discovered that the observer variation was greater than the variation between image types [3]. The authors recommended that “In mass survey work … all films be read independently by at least two interpreters”. Double reading in mammography and other types of radiologic screening is, however, not the purpose of the current study since the approach of the observer in screening work is different from that in clinical work. In screening, the focus leans towards finding true positives and avoiding false negatives, whereas in clinical work also false positive and true negative findings are of importance. Neither is the purpose of the current study the evaluation of double reading in a learning situation, such as the double reading of residents’ reports by specialists in radiology. In such cases, the report and findings of a resident are checked by a more experienced colleague. This has an educational purpose and serves to improve the final report to provide better healthcare, with a better patient outcome in the end. The value of such double reading is hardly debatable.

Double reading can be broadly divided into three categories: (1) both primary and secondary reading by radiologists of the same degree of sub-specialisation, in consensus, or serially with or without knowledge of the contents of the first report; (2) secondary reading by a radiologist of a higher level of sub-specialisation; (3) double reading of resident reports [5].

The concept of double reading is at times confusing and can apply to several practices.

In screening, the concept of double reading implies that if both readers are negative, the combined report is negative. If one or both readers are positive, the report is positive (i.e. the “Or” rule or “Believe the positive”). In dual reading, the two readers reach a consensus over the differing reports [6].

Some studies use arbitration: with conflicting findings, a third reader considers each specific disagreement and decides whether the reported finding is present or not. Similar to this is pseudo-arbitration: with conflicting findings, the independent and blinded report of a third reader casts the deciding “vote” in each dispute between the original readers. In contrast to the “true arbitration” model, the third reader is not aware of the specific disagreement(s) [7]. These concepts are summarised in Table 1.

**Table 1:** Various applications of single and double reading

First reader	Second reader	Third reader	Grouping	Type of double reading	Application	Included in review	Ref.
Specialist			Single reading	Single reading	Clinical practice	No	
CAD	Specialist		1st reader non-specialist	Single reader aided by CAD	Mammography, chest CT	No	[8]
Non-radiologist	Specialist			Report by other profession such as radiographer or clinician overseen by radiology specialist	Clinical practice	No	[9]
Resident	Specialist			Quality assurance	Teaching, clinical practice	No	[10]
Specialist	Specialist		2 readers	Independent reading; if one reader finds a lesion, the case is selected for further study, the OR rule	Screening	No	[3, 6]
Specialist	Specialist			Simultaneous reading to reach consensus	Clinical practice	Yes	[6]
Specialist	Specialist			Serially, blinded to other report	Research	Yes	[11]
Specialist	Specialist			Serially with knowledge of first report	Clinical practice	Yes	[12, 13]
Specialist	Specialist	Specialist	3rd reader arbitration	Arbitration; third reader considers each specific disagreement and decides	Quality assurance, research	Yes	[7]
Specialist	Specialist	Specialist		Pseudo-arbitration; third reader is not aware of the disagreements	Research	Yes	[7]
Specialist	Sub-specialist		Sub-specialist over-reading	Second reading with higher degree of sub-specialisation	Clinical practice	Yes	[5]

*CAD* computer aided diagnosis

Considering the paucity of evidence either for or against double reading among peers in clinical practice, the purpose of the current study was to, through a systematic review of available literature, gather evidence for or against double reading in imaging studies by peers and its potential value. A secondary aim was to evaluate double reading with the secondary reading being performed by a sub-specialist.

## Materials and methods

The study was registered in PROSPERO International prospective register of systematic reviews, CRD42017059013.

The inclusion criterion in the literature search was: studies calculating the rate of misses and overcalls with the aim of establishing the added value of double reading by human observers. The exclusion criteria were: (1) articles dealing solely with mammography; (2) articles dealing solely with screening; (3) articles dealing solely with double reading of residents; (4) articles not dealing with double reading; (5) reviews, editorials, comments, abstracts or case reports; (6) articles without abstract; (7) article not written in English, German, French or the Nordic languages; (8) duplicate publications of the same data.

### Literature search

A literature search was performed on 26 January 2017 in PubMed/MEDLINE and Scopus. The search expressions were a combination of “radiography, computed tomography (CT), magnetic resonance imaging (MRI) and double reading/reporting/interpretation” (Appendix 1).

Both authors read all titles and abstracts independently. All articles that at least one reviewer considered worth including were chosen for reading of the full text. After independent reading of the full text, articles fulfilling the inclusion criteria were selected. Disagreements were solved in consensus. The material was stratified into two groups depending on whether the double reading was performed by a colleague of similar or higher sub-specialty.

## Results

**Fig. 1:**
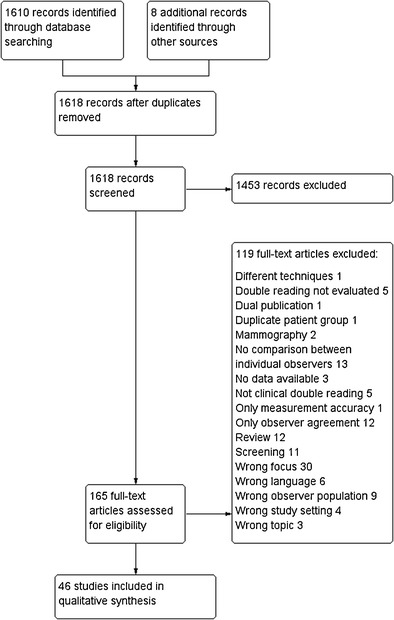
Study flow diagram

The literature search resulted in 1,610 hits. Another eight articles were added after manual perusal of the reference lists. Of these, 165 articles were chosen for reading of the full text. Forty-six of these that fulfilled the inclusion criteria and did not comply with the exclusion criteria were selected for final analysis. The study flow diagram is shown in Fig. 1. Study characteristics and results are shown in Table 2. Excluded articles are shown in Appendix 2.

**Table 2:** Study characteristics and results

First author, country	Year	Clinical setting	Method	Total number of cases	Results	Conclusion
Double reading by peers; CT						
Yoon LS, USA [13]	2002	Abdominal and pelvic trauma CT	Original report reviewed by a second non-blinded reader	512	30% discordant readings, patient care was changed in 2.3%	Most discordant readings do not result in change in patient care
Agostini C, France [14]	2008	CT in polytrauma patients	Official interpretation reviewed by two radiologists	105	280 lesions out of 765 (37%) were not appreciated during first reading, of these 31 major	Double reading is recommended in polytrauma patients
Sung JC, USA [15]	2009	Trauma CT from outside hospital	Re-interpretation by local radiologist	206	12% discrepancies, judged as perceptual in 26% and interpretive in 70%	Double reading is beneficial
Eurin M, France [16]	2012	Whole-body trauma CT	Scans were re-interpreted for missed injuries by second reader, blinded to initial data	177	157 missed injuries in 85 patients (48%), predominantly minor and musculoskeletal	Double reading is recommended
					The second reader missed injuries in 14 patients	
Bechtold RE, USA [17]	1997	Abdominal CT	Clinical report compared with reference standard from a consensus panel	694	56 errors in 694 patients	7.6% errors in CT abdomen, 2.7% clinically significant
Fultz PJ, USA [18]	1999	CT of ovarian cancer	Four independent readers tested single, single with checklist, paired consensus, and replicated readings	147	Sensitivity for single reader, checklist, paired and replicated readings were 93 to 94% with specificities 79, 80, 82 and 85%, almost all non-significant	The diagnostic aids did not lead to an improved mean observer performance, however an increase in the mean specificity occurred with replicated readings
Gollub MJ, USA [12]	1999	CT abdomen and pelvis in cancer patients	Original report and re-interpretation report by a non-blinded reader in another hospital was retrospectively compared	143	Major disagreement in 17%, treatment change in 3%	Reinterpretation of body CT scans can have a substantial effect on the clinical care
Johnson KT, USA [19]	2006	CT colonography with virtual dissection software	Single reading compared with double reading, no consensus	20	Sensitivity/specificity single reading 78–85/80–100%, sensitivity double reading 75–95%	5 mm polyps and larger. No significant increase in sensitivity with double reading
Murphy R, UK [20]	2010	CT colonography with minimal preparation	Independent and blinded double reading	186	Single reading found 11 cancers and double reading 12, at the expense of 5 false positives for single and 10 for double reading, giving positive predictive values of 69% and 54%, respectively	There is some benefit of double reporting; however, with major resource implications and at the expense of increased false-positives
Lauritzen PM, Norway [21]	2016	Abdominal CT	Double reading, peer review	1,071	Clinically important changes in 14%	Primary reader chose which studies should be double-read, thus probably more difficult cases. Important changes were made less frequently when abdominal radiologists were first readers, more frequently when they were second readers, and more frequently to urgent examinations
Wormanns D, Germany [8]	2004	Low-dose chest CT for pulmonary nodules	Independent double reading	9 patients with 457 nodules	Sensitivity of single reading, 54%; double reading, 67%; single reader with CAD, 79%. False positives, 0.9–3.9% for readers, 7.2% for CAD	Double reading and CAD increased sensitivity, CAD more than double reading, at the cost of more false positives for CAD
Rubin GD, USA [22]	2005	Pulmonary nodules on CT	Independent reading by three radiologists, reference standard by two thoracic radiologists + CAD	20	Sensitivity single reading 50%, double reading 63%, single reading + CAD 76–85%	Double reading increased sensitivity slightly. Inclusion of CAD increased sensitivity further
Wormanns D, Germany [23]	2005	Chest CT for pulmonary nodules	Independent double reading of low- and standard-dose CT	9 patients with 457 nodules	Sensitivity of single reading, 64%; double reading, 79%; triple reading, 87% (low-dose CT)	Double reading significantly increased sensitivity
						5-mm slices used in the study
Lauritzen PM, Norway [24]	2016	Chest CT	Double reading, peer review	1,023	Clinically important changes in 9%	Primary reader chose which studies should be double-read, thus probably more difficult cases. More clinically important changes were made to urgent examinations, chest radiologists made more clinically important changes than the other consultants
Lian K, Canada [25]	2011	CT angiography of the head and neck	Blinded double reading by two neuroradiologists in consensus, compared with original report by a neuroradiologist	503	26 significant discrepancies were found in 20 cases, overall miss rate of 5.2%	Double reading may decrease the error rate
Double reading by peers; radiography						
Markus JB, Canada [26]	1990	Double-contrast barium enema	Double and triple reporting, colonoscopy as reference standard	60	Sensitivity/specificity of single reading, 68/96%; double reading. 82/91%	Double reading increased sensitivity and reduced specificity slightly
Tribl B, Austria [27]	1998	Small-bowel double contrast barium examination in known Crohn’s disease	Clinical report double read by two gastrointestinal radiologists; ileoscopy as reference standard	55	Sensitivity/specificity of single reading, 66/82%; double reading. 68/91%	Negligible improvement by double reading
Canon CL, USA [28]	2003	Barium enemas, double- and single-contrast	Two independent readers, final diagnosis by consensus. Endoscopy as reference standard	994	Sensitivity/specificity of single reading, 76/91%; simultaneous dual reading, 76/86%	Dual reading led to an increased number of false positives which reduced specificity. No benefit in sensitivity
Marshall JK, Canada [29]	2004	Small-bowel meal with pneumocolon for diagnosis of ileal Crohn’s disease	Double reading of clinical report by two gastrointestinal radiologists with endoscopy as reference standard	120	Sensitivity/specificity of single reading, 65/90%; double reading, 81/94%	Possibly increased sensitivity with double reading, however unclear information on how study was performed
Hessel SJ, USA [7]	1978	Chest radiography	Independent reading by eight radiologists, combined by various strategies	100		Pseudo-arbitration was the most effective method overall, reducing errors by 37%, increasing correct interpretations 18%, and adding 19% to the cost of an error-free interpretation
Quekel LGBA, Netherlands [6]	2001	Chest radiography	Independent and blinded double reading as well as dual reading in consensus	100	Sensitivity/specificity of single reading, 33/92%; independent double reading, 46/87%; simultaneous dual reading, 37/92%	Double or dual reading increased sensitivity and decreased specificity, altogether little impact on detection of lung cancer in chest radiography
Robinson PJA, UK [30]	1999	Skeletal, chest and abdominal radiography in emergency patients	Independent reading by three radiologists	402	Major disagreements in 5–9% of cases	The magnitude of interobserver variation in plain film reporting is considerable
Soffa DJ, USA [31]	2004	General radiography	Independent double reading by two radiologists	3,763	Significant disagreement in 3%	Part of a quality assurance program
Double reading by peers; mixed modalities						
Wakeley CJ, UK [32]	1995	MR imaging	Double reading by two radiologists. Arbitration in case of disagreement	100	9 false-positive, 14 false-negative reports in 100 cases	The study promotes the benefits of double reading MRI studies
Siegle RL, USA [33]	1998	General radiology in six departments, including CT, nuclear medicine and ultrasound	Double reading by a team of QC radiologists	11,094	Mean rate of disagreement 4.4% in over 11,000 images	Rates of disagreement lower than previously reported
Warren RM, UK [34]	2005	MR breast imaging	Blinded and independent double reading by two observers, 44 in total!	1,541	Sensitivity/specificity of single reading, 80/88%; double reading, 91/81%	Double reading increased sensitivity at the cost of decreased specificity
Babiarz LS, USA [35]	2012	Neuroradiology cases	Original report by neuroradiologist, double reading by another neuroradiologist	1,000	2% rate of clinically significant discrepancies	Low rate of disagreements, but all worked in the same institution
Agrawal A, India [36]	2017	Teleradiology emergency radiology	Parallel dual reporting	3,779	3.8% error rate, CT abdomen and MRI head/spine most common error sources	Focused double read of pre-identified complex, unfamiliar or error-prone case types may be considered for optimum utilisation of resources
Harvey HB, USA [37]	2016	CT, MRI and ultrasound	Peer review using consensus-oriented group review	11,222	Discordance in 2.7%, missed findings most common	Highest discordance rates in musculoskeletal and abdominal divisions
Double reading by sub-specialist; abdominal imaging						
Kalbhen CL, USA [38]	1998	Abdominal CT for pancreatic carcinoma	Original report reviewed by sub-specialty radiologists	53	32% discrepancies in 53 patients, all under-staging	Reinterpretation of outside abdominal CT was valuable for determining pancreatic carcinoma resectability
Tilleman EH, Netherlands [39]	2003	CT or ultrasound in patients with pancreatic or hepatobiliary cancer	Reinterpretation by sub-specialised abdominal radiologist	78	48% of ultrasound and 30% of CT studies were judged as not sufficient for reinterpretation	Change in treatment strategy in 9%. Many initial reports were incomplete
					Major discordance in 8% for ultrasound, 12% for CT	
Bell ME, USA [40]	2014	After-hours body CT	Abdominal imaging radiologists reviewed reports by non-sub-specialists	1,303	4.4% major discrepancies in 742 cases double read by primary members of the abdominal imaging division, 2.0% major discrepancies in 561 cases double read by secondary members	The degree of sub-specialisation affects the rate of clinically relevant and incidental discrepancies
Lindgren EA, USA [5]	2014	CT, MR and ultrasound from outside institutions submitted for secondary interpretation	Second opinion by sub-specialised GI radiologist	398	5% high clinical impact and 7.5% medium clinical impact discrepancies	The second reader had 2% medium clinical impact discrepancies. There was a trend towards overcalls in normal cases and misses in complicated cases with pathology
Wibmer A, USA [41]	2015	Diagnosis of extracapsular extension of prostate cancer on MRI	Second-opinion reading by sub-specialised genitourinary oncological radiologists	71	Disagreement between the initial report and the second-opinion report in 30% of cases, second-opinion correct in most cases	Reinterpretation by sub-specialist improved detection of extracapsular extension
Rahman WT, USA [42]	2016	Abdominal MRI in patients with liver cirrhosis	Re-interpretation by sub-specialised hepatobiliary radiologist	125	10% of subjects had a discrepant diagnosis of hepatocellular cancer, and 10% of subjects had discrepant Milan status for transplant	Reinterpretations were more likely to describe imaging findings of cirrhosis and portal hypertension and more likely to make a definitive diagnosis of HCC
						50% change in management
Double reading by sub-specialist; chest						
Cascade PN, USA [43]	2001	Chest radiography	Performance of chest faculty and non-chest radiologists was evaluated	485,661	No difference in total rate of incorrect diagnoses, but non-chest faculty had a statistically significant higher rate of seemingly obvious misdiagnoses	There are several potential biases in the study which complicate the conclusions
Nordholm-Carstensen A, Denmark [44]	2015	Chest CT in colorectal cancer patients, classification of indeterminate nodules	Second opinion by sub-specialised thoracic radiologist	841	Sensitivity/specificity primary reading 74/99%, sub-specialist 92/100%	Higher sensitivity for the thoracic radiologist with fewer indeterminate nodules
Double reading by sub-specialist; neuro						
Jordan MJ, USA [45]	2006	Emergency head CT	Original report reviewed by sub-specialty neuroradiologists	1,081	4 (0.4%) clinically significant and 10 insignificant errors	Double reading of head CT by sub-specialist appears to be inefficient
Briggs GM, UK [46]	2008	Neuro CT and MR	Second opinion by sub-specialised neuro-radiologist	506	13% major discrepancy rate	The benefit of a formal specialist second opinion service is clearly demonstrated
Zan E, USA [47]	2010	Neuro CT and MR	Reinterpretation by sub-specialised neuroradiologist	4,534	7.7% of clinically important differences	Double reading is recommended
					When reference standards were available, the second-opinion consultation was more accurate than the outside interpretation in 84% of studies	
Jordan YJ, USA [48]	2012	Head CT, stroke detection	Original report reviewed by sub-specialty neuroradiologists	560	0.7% rate of clinically significant discrepancies	Low rate of discrepancies and double reading by sub-specialist was reported as inefficient. However the study was limited to ischaemic non-haemorrhagic disease
Double reading by sub-specialist; paediatric						
Eakins C, USA [49]	2012	Paediatric radiology	Cases referred to a children’s hospital were reviewed by a paediatric sub-specialist	773	22% major disagreements	Interpretations by sub-specialty radiologists provide important clinical information
					When final diagnosis was available, the second interpretation was more accurate in 90% of cases	
Bisset GS, USA [50]	2014	Paediatric extremity radiography	Official interpretation reviewed by one paediatric radiologist, blinded to official report. Arbitration by a second radiologist when reports differed	3,865	Diagnostic errors in the form of a miss or overcall occurred in 2.7% of the radiographs	Diagnostic errors quite rare in paediatric extremity radiography. Clinical significance of the discrepancies was not evaluated
Onwubiko C, USA [51]	2016	CT abdomen in paediatric trauma patients	Re-review of images by paediatric radiologist	98	12.2% new injuries identified, 3% had solid organ injuries upgraded, and 4% downgraded to no injury	Clear benefit to having referring hospital trauma CT scans reinterpreted by paediatric radiologists
Double reading by sub-specialist; other applications						
Loevner LA, USA [52]	2002	CT and MR in head and neck cancer patients	Second opinion by sub-specialised neuroradiologist	136	Change in interpretation in 41%, TNM change in 34%, mostly up-staging	Sub-specialist increases diagnostic accuracy
Kabadi SJ, USA [53]	2017	CT, MR and ultrasound from outside institutions submitted for formal over-read	Retrospective review	362	12.4% had clinically significant discrepancies	64% perceptual errors
						Strategies for reducing errors are suggested

*CAD* computer aided diagnosis, *HCC* hepatocellular cancer

When perusing the material, it was found that there were not sufficient data to perform a meta-analysis. Instead, a verbal summary was performed. In the results, two distinct groups of studies appeared: studies reporting double reading by peers of similar competence level and studies reporting the second reading performed by a sub-specialist, often performed at a referral hospital.

### Double reading by peers of similar degree of sub-specialisation

Fifteen articles evaluated double reading in CT.

- In trauma CT, three papers found initial discordant readings of 26–37% [13–15]. However, in one of these articles patient care was changed in only 2.3% by a non-blinded second reader [13]. Eurin et al. [16] reported a high rate of missed injuries initially, predominantly minor and musculoskeletal injuries.
- In abdominal CT, a discrepancy rate of 17% resulted in 3% treatment change when reviewed by a non-blinded second reader [12]. Five articles evaluated sensitivity and specificity. In CT of ovarian cancer and CT colonography, there was a non-significant trend towards higher sensitivity in double reading [18, 19], but double reading increased the false-positive rate [20].
- In chest CT for pulmonary nodules, double reading increased sensitivity [8, 22, 23], but computer-aided diagnosis (CAD) was even more beneficial [8, 22]. Another article found clinically important changes in 9% of cases [24].

Eight articles evaluated double reading in radiography.

- Two articles found negligible improvement by double reading in small-bowel and large-bowel barium studies, one study even reported increased false positives with double reading [27, 28].
- In chest radiography, Hessel et al. [7] combined independent readings by eight radiologists. Using a third independent interpretation to resolve disagreements between pairs of readers (pseudo-arbitration) was the most effective method overall, reducing errors by 37%, increasing correct interpretations by 18%, and adding 19% to the cost of an error-free interpretation.
- Quekel et al. [6] reported that double or dual reading increased sensitivity, at the same time reducing specificity.
- Two articles quoted 3–9% disagreement between observers in general radiography [30, 31].

Mixed modalities.

- Siegle et al. [33] evaluated general radiology in six departments, and found a mean rate of disagreement of 4.4%.
- In another large study, 11,222 cases (3.3% of the total production) underwent randomised peer review using a consensus-oriented group review with a rate of discordance (“report should change”) of 2.7% [37].
- Babiarz and Yousem [35] found 2% disagreement when 1,000 neuroradiology cases were double read by another neuroradiologist, all working in the same institution.
- In breast MRI, double reading increased sensitivity from 80 to 91%, while reducing specificity from 88 to 81% [34].
- Agrawal et al. [36] performed parallel dual reporting in teleradiology emergency radiology which resulted in 3.8% disagreements. The authors suggested that abdominal CT and head/spine MRI were the most common error sources and that a focused double reading of error-prone case types may be considered for optimum utilisation of resources.

### Second reading by a sub-specialist

- Six articles reported on abdominal imaging, five of these for distinct conditions, usually malignancy. The discrepancy rates for these varied from about 12% up to 50% [5, 38, 39, 41, 42].
- Bell and Patel [40] reported on 1,303 cases of body CT with the primary report from non-sub-specialised radiologists and found a higher frequency of clinically relevant discrepancies in the 742 cases that were double read by radiologists with a higher degree of sub-specialisation.
- In chest radiography, a statistically significantly higher rate of seemingly obvious misdiagnoses was found for non-chest speciality radiologists [43], while a thoracic radiologist had higher sensitivity and reported fewer indeterminate nodules in chest CT for colorectal cancer [44].
- In neuroradiology, two articles demonstrated the benefit from sub-specialist second opinion [46, 47], while two did not [45, 48].
- In paediatric radiology, Eakins et al. [49] found a high rate of discrepancies in neuroimaging and body studies, while discrepancies were much rarer in extremity radiography [50]. In abdominal trauma CT, 12 new injuries were found in 98 patients [51].

## Discussion

This systematic review found a wide range of significant discrepancy rates, from 0.4 to 22%, with minor discrepancies being much more common. Most of this variability is probably due to study setting. Double reading generally increased sensitivity at the cost of decreased specificity. One area where double reading seems to be important is in trauma CT, which is not surprising considering the large number of images and often stressful conditions under which the primary reading is performed. Thoracic and abdominal CT were also associated with more discrepancies than head and spine CT [54]. Higher rates of discrepancy can be expected in cases with a high probability of disease with complicated imaging findings [5].

More surprising was the fact that double reading by a sub-specialist almost invariably changed the initial reports to a high degree, although the second reader was also the reference standard for the study, which might have introduced bias. This leads to the conclusion that it might be more efficient to strive for sub-specialised readers than to implement double reading. It might also be more cost-efficient considering the fact that in one study, double reading of one-third of all studies consumed an estimated 20–25% of all working hours in the institutions concerned [1]. In modern digital radiology it is easy to send images to another hospital, and it should thus be possible to include even small radiology departments in a large virtual department where all radiologists can be sub-specialised. However, even a sub-specialised reader is subject to the same basic reading errors and this needs further study comparing outcomes from various reading strategies.

The primary goal of the current study was to evaluate double reading in a clinically relevant context, i.e. where the second reader double-reads the case in a non-blinded context before the report is finalised. Only two studies used a method approaching this [12, 13]. Reinterpretation of body CT in another hospital was beneficial [12] but double reading of abdominal and pelvic trauma CT resulted in only 2.3% changes in patient care [13].

One method for peer review of radiology reports is error scoring such as is practiced in the RadPeer program [55]. This differs from clinical double reading in that it does not confer direct benefit for the patient at hand. The use of old reports can also be seen as a form of second reading [56].

Double reading has been evaluated in a recent systematic review which dedicated much space to mammography screening [57]. This review suggested further attention to other common examinations and implementation of double reading as an effective error-reducing technique. This should be coupled with studies on its cost-effectiveness. The literature search in the current study resulted in some additional articles and a slightly different conclusion, which is not surprising considering the wide variety of studies included. In a systematic review on CT diagnosis, a major discrepancy rate of 2.4% was found, even lower when the secondary reader was non-blinded [54]. There is also a Cochrane review on audit and feedback which borders on the subject in the current study, even though no radiology-specific articles were included [58]. Errors and discrepancies in radiology have been covered in a recent review article [59].

Observer variation analysis is now customary when evaluating imaging modalities or procedures, or when starting studies on larger image materials [60–62], and it is well known that observer variation can be small or large between observers, due to differences in experience and variations in image quality or ease of detection and characterisation of a lesion.

A quality assessment of the individual evaluated articles was not performed in the current study. It was judged to be not feasible to get any meaningful results out of this, due to the wide variability in subject matter and methods.

Limitations of the study are the widely varying definitions of what is a clinically important discrepancy, which makes a meaningful meta-analysis impossible. In studies with a sub-specialised second reader there is a risk that the discrepancy rate is inflated since the second reader decides what should be included in the report.

In conclusion, the systematic review found, in general, rather low discrepancy rates when double-reading radiological studies. The benefit of double reading must be balanced by the considerable number of working hours a systematic double reading scheme requires. A more profitable scheme might be to use systematic double reading for selected, high-risk examination types. A second conclusion is that there seems to be a value in sub-specialisation for increased report quality. A consequent implementation of this would have far-reaching organisational effects.

## Electronic supplementary material

ESM 1 (DOCX 82 kb)

ESM 2 (DOCX 24 kb)
