# Supplementary material for: Added value of double reading in diagnostic radiology,a systematic review
Source: Insights Imaging. 2018 Mar 28;9(3):287–301. doi: 10.1007/s13244-018-0599-0 (PMC5990995; doi:10.1007/s13244-018-0599-0)
Supplement: Supplementary file 1 — (DOCX 82 kb) [file 13244_2018_599_MOESM1_ESM.docx]

**Appendix 1. Search expressions**

PubMed/MEDLINE:

((((((((radiography OR radiology))) OR ((Tomography, X-Ray Computed [MeSH] OR “computed tomography” OR “computerized tomography” OR “computer tomography” OR CT))) OR ((Magnetic Resonance Imaging [MeSH] OR “magnetic resonance” OR MRI))) OR ((Image Interpretation, Computer-Assisted [MeSH] OR Diagnosis, Computer-Assisted [MeSH] OR CAD)))) AND ((((((“double interpretation” OR “dual interpretation” OR “triple interpretation” OR “multiple interpretation”))) OR ((“double reporting” OR “dual reporting” OR “triple reporting” OR “multiple reporting”))) OR ((“double reading” OR “dual reading” OR “triple reading” OR “multiple reading”))) OR ((“second reading” OR reinterpretation OR “peer review” OR “peer feedback”))))

Scopus:

( ( ( ( TITLE-ABS-KEY ( radiography ) ) OR ( TITLE-ABS-KEY ( radiology ) ) ) OR ( ( TITLE-ABS-KEY ( x-ray computed tomography ) ) OR ( TITLE-ABS-KEY ( computed tomography ) ) OR ( TITLE-ABS-KEY ( computerized tomography ) ) OR ( TITLE-ABS-KEY ( ct ) ) ) OR ( ( TITLE-ABS-KEY ( magnetic resonance imaging ) ) OR ( TITLE-ABS-KEY ( magnetic resonance ) ) OR ( TITLE-ABS-KEY ( mri ) ) ) ) ) AND ( ( ( TITLE-ABS-KEY ( "double interpretation" OR "dual interpretation" OR "triple interpretation" OR "multiple interpretation" ) ) OR ( TITLE-ABS-KEY ( "double reporting" OR "dual reporting" OR "triple reporting" OR "multiple reporting" ) ) OR ( TITLE-ABS-KEY ( "double reading" OR "dual reading" OR "triple reading" OR "multiple reading" ) ) ) )
