# Supplementary material for: Added value of double reading in diagnostic radiology,a systematic review
Source: Insights Imaging. 2018 Mar 28;9(3):287–301. doi: 10.1007/s13244-018-0599-0 (PMC5990995; doi:10.1007/s13244-018-0599-0)
Supplement: Supplementary file 2 — (DOCX 24 kb) [file 13244_2018_599_MOESM2_ESM.docx]

**Appendix 2. Excluded studies after full-text reading**

| **Author** | **Year** | **Reason for exclusion** |
| --- | --- | --- |
| Anonymous [63] | 1955 | Screening |
| Griep WA [64] | 1955 | Screening |
| Yerushalmy J [65] | 1955 | Review |
| Williams RG [66] | 1958 | Screening |
| Discher DP [67] | 1971 | Wrong focus |
| Felson B [68] | 1973 | Screening |
| Angerstein W [69] | 1975 | Only observer agreement |
| Herman PG [70] | 1975 | Wrong focus |
| Labrune M [71] | 1976 | No comparison between individual observers |
| Stitik FP [72] | 1978 | No data available |
| Aoki M [73] | 1985 | Wrong language |
| Gjorup T [74] | 1985 | Only observer agreement |
| Gjorup T [75] | 1986 | Only observer agreement |
| Fukuhisa K [76] | 1989 | Wrong language |
| Stephens S [77] | 1989 | Wrong focus |
| Shaw NJ [78] | 1990 | Only observer agreement |
| Anderson N [79] | 1991 | Not clinical double reading |
| Corbett SS [80] | 1991 | Only observer agreement |
| Haug PJ [81] | 1991 | Wrong focus |
| Hopper KD [82] | 1991 | Wrong focus |
| Slovis TL [83] | 1991 | No comparison between individual observers |
| Matsumoto T [84] | 1992 | Wrong language |
| Frank MS [85] | 1993 | Wrong focus |
| O'Shea TM [86] | 1993 | Wrong focus |
| Friedman DP [87] | 1995 | Wrong focus |
| Gacinovic S [88] | 1996 | Only observer agreement |
| Nitowski LA [89] | 1996 | Wrong observer population |
| Filippi M [90] | 1997 | No comparison between individual observers |
| Gale ME [91] | 1997 | Different techniques |
| Law RL [92] | 1999 | No comparison between individual observers |
| Jiang Y [93] | 2000 | Mammography |
| Kopans DB [94] | 2000 | Review |
| Connolly DJA [95] | 2002 | Double reading not evaluated |
| Fidler JL [96] | 2002 | Not clinical double reading |
| Leslie A [97] | 2002 | No comparison between individual observers |
| Murphy M [98] | 2002 | Wrong observer population |
| Summers RM [99] | 2002 | No comparison between individual observers |
| Baarslag HJ [100] | 2003 | Only observer agreement |
| Johnson CD [101] | 2003 | Dual publication |
| Quekel LGBA [102] | 2003 | Wrong language |
| Borgstede JP [103] | 2004 | Wrong topic |
| Halsted MJ [104] | 2004 | Review |
| Jarvenpaa R [105] | 2004 | Wrong observer population |
| Johnson CD [106] | 2004 | Screening |
| Smith PD [107] | 2004 | Wrong focus |
| Taylor P [108] | 2004 | Mammography |
| Barnhart HX [109] | 2005 | Wrong topic |
| Booth AM [110] | 2005 | Only observer agreement |
| Bradley AJ [111] | 2005 | Wrong observer population |
| Den Boon S [112] | 2005 | Screening |
| Jarvenpaa R [113] | 2005 | Duplicate patient group |
| Peldschus K [114] | 2005 | No comparison between individual observers |
| Birnbaum LM [115] | 2006 | Wrong observer population |
| Borgstede J [116] | 2007 | Review |
| Foinant M [117] | 2007 | Double reading not evaluated |
| Fraioli F [118] | 2007 | Screening |
| Capobianco J [119] | 2008 | Wrong language |
| Johnson CD [120] | 2008 | Wrong focus |
| Law RL [9] | 2008 | Wrong observer population |
| Law RL [121] | 2008 | Wrong observer population |
| Jackson VP [55] | 2009 | Wrong focus |
| Nellensteijn DR [122] | 2009 | Wrong study setting |
| Brinjikji W [123] | 2010 | Only observer agreement |
| Liu PT [124] | 2010 | Wrong study setting |
| Monico E [125] | 2010 | Wrong focus |
| Saurin JC [126] | 2010 | Not clinical double reading |
| Sheu YR [127] | 2010 | Wrong focus |
| Brook OR [128] | 2011 | Wrong focus |
| Husby JA [2] | 2011 | No data available |
| Ornetti P [62] | 2011 | Only measurement accuracy |
| Provenzale JM [129] | 2011 | Wrong focus |
| Sasaki Y [130] | 2011 | Screening |
| Bender LC [131] | 2012 | Wrong topic |
| Hussain S [132] | 2012 | No data available |
| McClelland C [133] | 2012 | Wrong study setting |
| Scaranelo AM [134] | 2012 | Wrong focus |
| Swanson JO [135] | 2012 | Wrong focus |
| Wang Y [136] | 2012 | Screening |
| Zhao Y [137] | 2012 | Not clinical double reading |
| Butler GJ [138] | 2013 | Review |
| d'Othee BJ [139] | 2013 | Double reading not evaluated |
| Gunn AJ [140] | 2013 | Wrong focus |
| Iussich G [141] | 2013 | No comparison between individual observers |
| Iyer RS [142] | 2013 | No comparison between individual observers |
| O'Keeffe MM [143] | 2013 | Wrong focus |
| Pairon JC [144] | 2013 | No comparison between individual observers |
| Rana AK [145] | 2013 | No comparison between individual observers |
| Sun H [146] | 2013 | No comparison between individual observers |
| Abujudeh H [147] | 2014 | Review |
| Alkasab TK [148] | 2014 | Review |
| Collins GB [149] | 2014 | Wrong observer population |
| Eisenberg RL [150] | 2014 | Double reading not evaluated |
| Garrett KG [10] | 2014 | Wrong observer population |
| Iussich G [151] | 2014 | No comparison between individual observers |
| Iyer RS [152] | 2014 | Wrong focus |
| Kanne JP [153] | 2014 | Review |
| Laurent F [154] | 2014 | Only observer agreement |
| Pairon JC [155] | 2014 | Wrong focus |
| Wu MZ [54] | 2014 | Review |
| Donnelly LF [156] | 2015 | Double reading not evaluated |
| Guerin G [11] | 2015 | Only observer agreement |
| Lauritzen PM [1] | 2015 | Wrong focus |
| Rosskopf AB [157] | 2015 | Wrong focus |
| Strickland NH [158] | 2015 | Review |
| Xu DM [159] | 2015 | Screening |
| Chung JH [160] | 2016 | Not clinical double reading |
| Grenville J [161] | 2016 | Wrong focus |
| Kruskal J [162] | 2016 | Wrong focus |
| Larson DB [163] | 2017 | Review |
| Lim HK [164] | 2016 | Wrong study setting |
| Maxwell AJ [165] | 2017 | Screening |
| Natarajan V [166] | 2017 | Wrong focus |
| O'Keeffe MM [167] | 2016 | Wrong focus |
| Olthof AW [168] | 2016 | Wrong focus |
| Pedersen MR [169] | 2016 | Only observer agreement |
| Pow RE [57] | 2016 | Review |
| Verma N [170] | 2016 | Wrong focus |
| Vural U [171] | 2016 | Wrong language |
| Steinberger S [172] | 2017 | Wrong focus |
